# An Aptamer-Based Capacitive Sensing Platform for Specific Detection of Lung Carcinoma Cells in the Microfluidic Chip

**DOI:** 10.3390/bios8040098

**Published:** 2018-10-20

**Authors:** Ngoc-Viet Nguyen, Chun-Hao Yang, Chung-Jung Liu, Chao-Hung Kuo, Deng-Chyang Wu, Chun-Ping Jen

**Affiliations:** 1 Department of Mechanical Engineering, National Chung Cheng University, 621 Chia Yi, Taiwan; vietnn.mt@gmail.com (N.-V.N.); milkk1031@gmail.com (C.-H.Y.); 2 Division of Gastroenterology, Department of Internal Medicine, Kaohsiung Medical University Hospital, 807 Kaohsiung, Taiwan; 1020590@ms.kmuh.org.tw (C.-J.L.); kjh88kmu@gmail.com (C.-H.K.); dechwu@yahoo.com (D.-C.W.); 3 Center for Stem Cell Research, Kaohsiung Medical University, 807 Kaohsiung, Taiwan; 4 Institute of Biomedical Science, National Sun Yat-sen University, 804 Kaohsiung, Taiwan; 5 Division of Internal Medicine, Kaohsiung Municipal Hsiao-Kang Hospital, Kaohsiung Medical University, 807 Kaohsiung, Taiwan

**Keywords:** aptamer, lung cancer, self-assembly, impedance measurement, capacitive sensor

## Abstract

Improvement of methods for reliable and early diagnosis of the cellular diseases is necessary. A biological selectivity probe, such as an aptamer, is one of the candidate recognition layers that can be used to detect important biomolecules. Lung cancer is currently a typical cause of cancer-related deaths. In this work, an electrical sensing platform is built based on amine-terminated aptamer modified-gold electrodes for the specific, label-free detection of a human lung carcinoma cell line (A549). The microdevice, that includes a coplanar electrodes configuration and a simple microfluidic channel on a glass substrate, is fabricated using standard photolithography and cast molding techniques. A procedure of self-assembly onto the gold surface is proposed. Optical microscope observations and electrical impedance spectroscopy measurements confirm that the fabricated microchip can specifically and effectively identify A549 cells. In the experiments, the capacitance element that is dominant in the change of the impedance is calculated at the appropriate frequency for evaluation of the sensitivity of the biosensor. Therefore, a simple, inexpensive, biocompatible, and selective biosensor that has the potential to detect early-stage lung cancer would be developed.

## 1. Introduction

During the past three decades, many diseases in humans have emerged strongly, including cancer. Lung cancer is one of the most frequently-recognized cancers in both men and women, with over 1.5 million new cases occurring per year, accounting for about 13% of total cancer diagnoses [1]. The existing diagnosis methods, which are based on histological examinations of the suspicious tissue in the context of its clinical and morphological features [2], are often very expensive, and require advanced instruments. Moreover, they are not sensitive enough to diagnose the disease in its early stages, and are non-specific for cancer classification. Cancer cells can be found in many different states due to differences at the morphological and molecular levels [3,4]. The stages of cancer are closely related to the change of cells, such as cell morphology, proliferation, and differentiation. Hence, developing sensitive and specific approaches for the detection of cancerous cells is crucial. The A549 cell line, a typical carcinoma subtype of non-small cell lung cancer, has been known as the circulating tumor cell model of the early stage of cancer [5]. Enhancement of early detection and treatment of the A549 cells is therefore essential for reducing mortality rates.

Developments is microfluidics and nanotechnology (for example, the development of good indicators of the presence of a primary tumor) have improved the detection and capture capabilities of tumor cells [6,7]. Recent advances of noninvasive tests based on surface-specific probes have received significant attention for cancer diagnosis and for the identification of cancer subtypes. Several biosensors that use enzymes, receptors, and antibodies have been reported [8,9,10]. One of the main disadvantages of using antibodies is their instability due to irreversible denaturation. Aptamers are single-stranded DNA or RNA oligonucleotides that have emerged as an alternative approach for specific target recognition expressed on the surface membranes, with high affinity and selectivity [11]. Aptamer types are isolated through a selection process known as SELEX (systematic evolution of ligands by exponential enrichment). Several studies relating to the aptamer-based biosensors for the detection of proteins [12,13], enzymes [14], molecules [15,16], viruses [17], antibiotics [18], and cancer cells [19,20,21,22] have been explored. An aptamer-coated silicon nanowire substrates for capturing circulating tumor cells from blood samples was developed [23]. The device was capable of specifically capturing A549 cells with over 90% efficacy. Another biosensor based on a MCU1 aptamer attached onto gold nanoparticles was also designed for the sensitive and selective detection of A549 cell [24]. The sensor showed a high affinity for non-small lung cancer cells (A549) compared with the other control cancer cells, including human prostate (PC3), normal lung (MRC-5), and liver tumor (HepG2) cells. Aptamer molecules show several distinctive advantages because of their unique binding properties. They are often stable in harsh biological environments, preserve their structures at high temperatures, and can be easily produced in bulk [11].

In recent years, electrical impedance-based approaches have been gaining much attention in biosensor research [25,26,27]. This type of sensor has many advantages, such as simplicity, miniaturizability, fast analysis, sensitive response, low cost, and suitability for integrated microsystems [28,29,30]. One example is electrical impedance spectroscopy (EIS), which is a label-free technique that allows for the determination of the biological medium changes between the electrodes by measuring their interfacial capacitance and resistance [31,32,33,34,35]. A silicon nanowire-based cell impedance sensor was developed to monitor the spreading-induced electrical differences between cancerous and normal lung cells [36]. This method takes rather a long time to make measurements and for the maintenance the culture conditions during cell growth process. A cheaper, faster, and simpler device with a circle-on-line microelectrodes structure was built for distinguishing lung cell lines using a dielectrophoretic impedance measurement method [37]. However, the examinations in these chips actually used a single type of cell samples, and did not involve any specific target cell selection from the blood. Aptasensors that combine aptamers and EIS have become a powerful method for the identification of the specific cells in many works [38,39,40,41,42]. Impedance changes may arise when the target proteins or cells bind to the receptor, becoming immobilized on the electrode surface, thereby displacing medium solution molecules. Many aptasensors were used in fabricating the conjugation of the aptamers with magnetic beads, nanostructures, nanoparticles, or nanomaterials to improve the surface-to-volume ratios and sensitivity [41,43,44,45]. However, the use of self-assembly on microelectrodes has the advantage of a simple surface immobilization process with high reproducibility and low cost [46]. DNA aptamer-modified gold electrodes were shown to be capable of detecting lung cancer-related proteins in crude blood plasma samples [47]. A sensitive and selective electrochemical sensor based on amine aptamer-functionalized graphite screen printed electrodes was constructed for the detection of colorectal cancer (CT26) cells [39].

In this study, the combination of DNA aptamers and impedance measurements have been utilized to build a simple microfluidic platform for the detection of the A549 human lung carcinoma cell line. A process of self-assembled monolayers (SAMs) of the gold surface was given. The probes for trapping target cells were prepared by the conjugation of the amino-labeled aptamers onto carboxylic acid functionalized gold electrodes. The efficiency of trapping cells was expressed by monitoring the change of the microscopic images. EIS was performed at frequencies ranging from 0.1 kHz to 1 MHz to demonstrate the binding events. The capacitive response of the impedance was investigated at different cell concentrations to evaluate the performance of the biosensor. The obtained results promise a powerful method for the identification of cancer cell lines with high affinity, selectivity, and specificity.

## 2. Materials and Methods

### 2.1. Chemicals and Reagents

Most of the chemicals, including DNA aptamer with 5′-thiol modification, thiol PEG carboxylic acid (HS-PEG-COOH), 1-ethyl-3-(3-dimethylaminopropyl)carbodiimide hydrochloride (EDC), N-hydroxysuccinimide (NHS), and phosphate-buffered saline solutions, were purchased from Sigma-Aldrich (St. Louis, MO, USA). The aptamer was provided in a dried form, with the sequence of 5′-ACGCT CGGAT GCCAC TACAG GGTTG CATGC CGTGG GGAGG GGGGT GGGTT TTATA GCGTA CTCAG CTCAT GGACG TGCTG GTGAC-3′—NH_2_ and selective binding to A549 lung cancer cells [48]. HS-PEG-COOH and EDC/NHS solutions were prepared in deionized water and 0.1 M MES buffer, respectively. The stock aptamer was dissolved completely to the desired concentration with a TE buffer (pH 8.0, 10 mM Tris, and 1 mM EDTA) and stored at −20 °C. The washing buffer was prepared by adding 5 mmol MgCl_2_ and 4.5 g glucose into 1 L of 10 mM PBS (PBS 1× at pH 7.4) without calcium and magnesium. The binding buffer was created by adding 0.1 mg tRNA and 1 mg bovine serum albumin (BSA, Sigma, St. Louis, MO, USA) to 1 mL washing buffer [48]. These buffers can be stored at 4 °C for up to 1 month. All aqueous solutions were diluted with deionized water (18.2 MΩ cm) from a Direct-Q system (Milli-Q, Millipore Simplicity, Billerica, MA, USA).

### 2.2. Microchip Design and Fabrication

**Figure 1:**
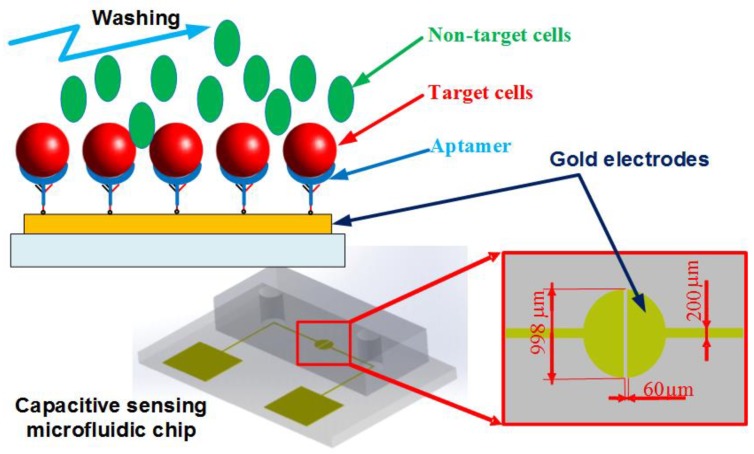
Schematic of the microfluidic chip. The aptamer-modified gold electrodes are utilized to capture the target cells, while the non-target cells are washed out of the channel. The impedance measurement could be performed within the chip to recognize the presence of the cells.

Figure 1 shows the dimensions and design of the proposed microfluidic chip. The microchip consists of a polydimethylsiloxane (PDMS) channel, a glass substrate, and a gold microelectrodes structure patterned on the glass surface.

**Figure 2:**
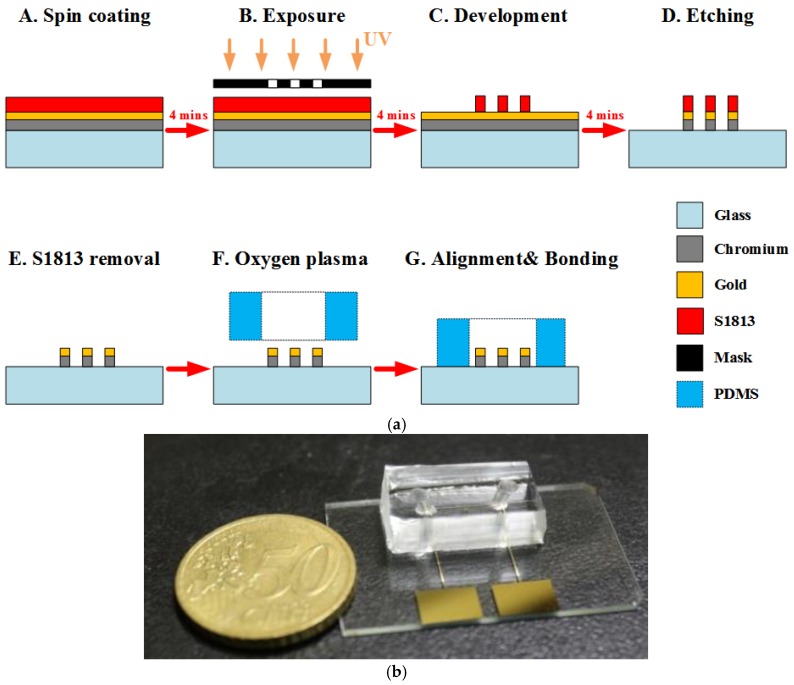
(**a**) The fabrication process of the chip. (**b**) Photograph of a fabricated microchip using the standard photolithography process.

In this work, a coplanar two-electrode configuration was used. The advantages are its simplicity of fabrication, and the ease with which it is possible to monitor the change of material properties inside the electric field between the electrodes based on electrode-solution interface impedance measurement. The electrode surface was designed to be large enough for the convenient observation of captured cells. The device was fabricated using a typical soft lithography procedure (Figure 2a), as reported previously [49]. First, the gold-glass side was cleaned with piranha solution (96% H_2_SO_4_: 30% H_2_O_2_ by the volume ratio of 3:1) for 30 min, and then was rinsed with deionized water. Subsequently, the photomask was aligned on the surface of the substrate coated with a layer of positive photoresist (Shipley 1813, MicroChem Co., Ltd., Westborough, MA, USA). The photoresist was then exposed via UV light to define the etching mask. Following a process of post-exposure baking, developing, hard baking, and wet etching the microelectrode structure onto glass substrate was finally performed.

Using a cast molding technique, a SU-8 (2050, MicroChem Corp., Newton, MA, USA) master mold with channel pattern on the surface of a silicon wafer was created. A degassed mixture of PDMS prepolymer and curing agent (Sylgard-184 Silicone Elastomer Kit, Dow Corning, Midland, MI, USA) at a weight ratio of 10:1 was poured onto the prepared master mold. Then, the PDMS block was baked at 75 °C for 2 h, and was released from the SU-8 mold after curing. Finally, the PDMS piece punched with fluidic ports was permanently bonded to the substrate using an oxygen plasma chamber (Model PDC-32G, Harrick Plasma Corp., Ithaca, NY, USA). The obtained channel height and width were approximately 50 μm and 1 mm, respectively. Figure 2b shows an image of the fabricated microchip.

### 2.3. Cell Preparation

Human epithelial adenocarcinoma cells including A549 (human non-small cell lung cancer cell line), Hela (human cervical cancer cell line), MKN45 (human gastric cancer cell line), and Caco-2 (human colorectal cancer cell line) were cultured for experimental demonstrations of the proposed microfabricated device. Minimum Essential medium (MEM), Dulbecco’s Modified Eagle Medium (DMEM), fetal bovine serum (FBS), L-Glutamine, and penicillin/streptomycin solutions for cell culture were purchased from Gibco (Grand Island, NY, USA). All tumor cells were incubated in a humidified atmosphere containing 5% carbon dioxide at 37 °C. The culture medium was replaced every 1 day to 2 days. Prior to the experiments, the cells were collected from the cell culture dishes by standard trypsinization. The cell samples were then washed three times by centrifugation in the buffer solution. The cell concentration and viability were assessed by trypan blue dye exclusion using a hemocytometer with two counting grids.

### 2.4. Aptamer on Self-Assembled Monolayers-Functionalized Gold Electrodes

**Figure 3:**
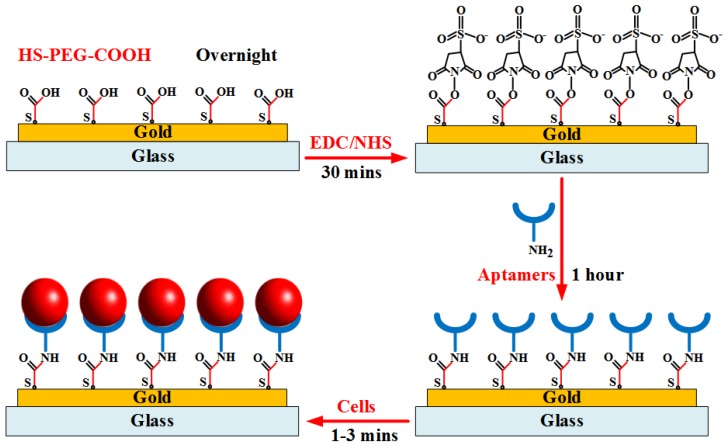
Illustration of aptamer immobilization procedure onto gold substrate for binding of target cells.

The layer-by-layer assembly surface procedure is illustrated in Figure 3. The gold substrate surfaces were first washed with the PBS washing buffer solution. The electrodes were covered with 0.1 mM HS-PEG-COOH solution and stored overnight at 4 °C from 12 h to 24 h. The gold–sulfur (Au–S) interaction formed between thiols and the gold surface provided the binding forces to generate robust SAMs for aptamer application. The modified electrodes were then continuously washed by the buffer solution. Next, the carboxylic groups on the electrode surfaces were activated in a solution containing 0.1 M NHS and 0.4 M EDC for 30 min to prepare stable amine-reactive esters of carboxylate groups for crosslinking with the amino-labeled aptamers. Subsequently, the initial 100 mM amine-labeled aptamers was diluted to a specific concentration using the binding buffer. The mixture solution was heated at 95 °C for 5 min. Then, tubes were left on the bench for 15 min at room temperature. The electrodes were washed again with the washing buffer, followed by the addition of refreshed aptamer solution onto the gold substrates for 1 h at room temperature. Finally, the cell sample at a given concentration was injected into the channel to bind target cells on the electrode surfaces. The channel was then washed by the buffer solution to remove nonspecific adsorbed biomolecules. The flow rate below 10 µL/min that was supplied into the microfluidic channel was applied to all experiments.

### 2.5. Apparatus

**Figure 4:**
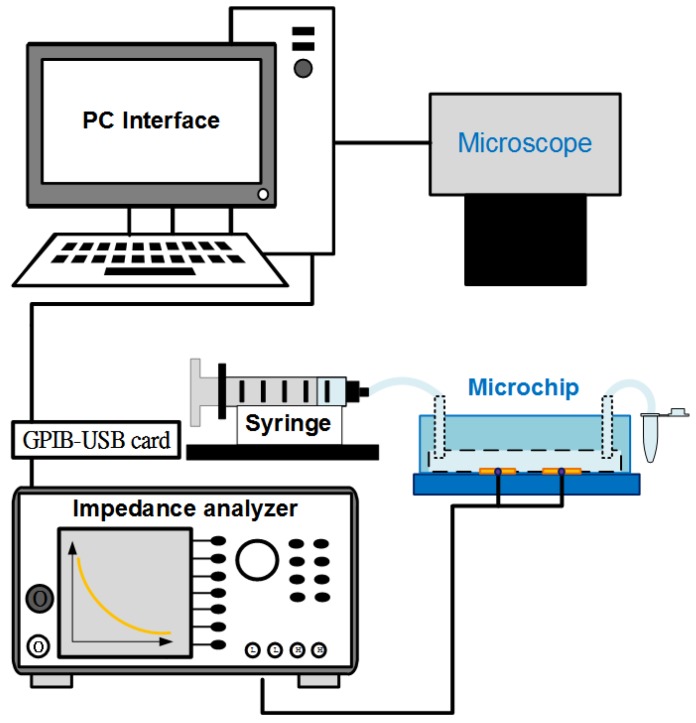
Diagram of experimental system using an inverted microscope and an impedance analyzer controlled with LabVIEW software interface.

The system for conducting experiments is schematically described in Figure 4. Electrochemical Impedance Spectroscopy (EIS) measurements were performed in a wide frequency range using an impedance analyzer (Wayne Kerr 6420, New Boston, TX, USA). The electrodes of the aptasensor were connected to the device by BNC cables. Used solutions were injected into the channel of the chip using a syringe pump (Model KDS 101, KD Scientific Inc., Holliston, MA, USA). The observations of cell samples were recorded using a fluorescence microscope (BX43, Olympus, Tokyo, Japan) with a mounted CCD camera (DP71, Olympus, Tokyo, Japan) connected to a computer running Olympus DP Controller image software. The electrical data were transferred to the computer via a digital interface (GPIB-USB-HS, National Instruments Corporation, Austin, TX, USA). The impedance parameters of the microchip were finally determined using LabVIEW software. All the impedance measurements were carried out at room temperature in the 10 mM PBS buffer solution.

## 3. Results and Discussion

### 3.1. Microscopic Responses of the Aptamer-Functionalized Gold Microelectrodes

**Figure 5:**
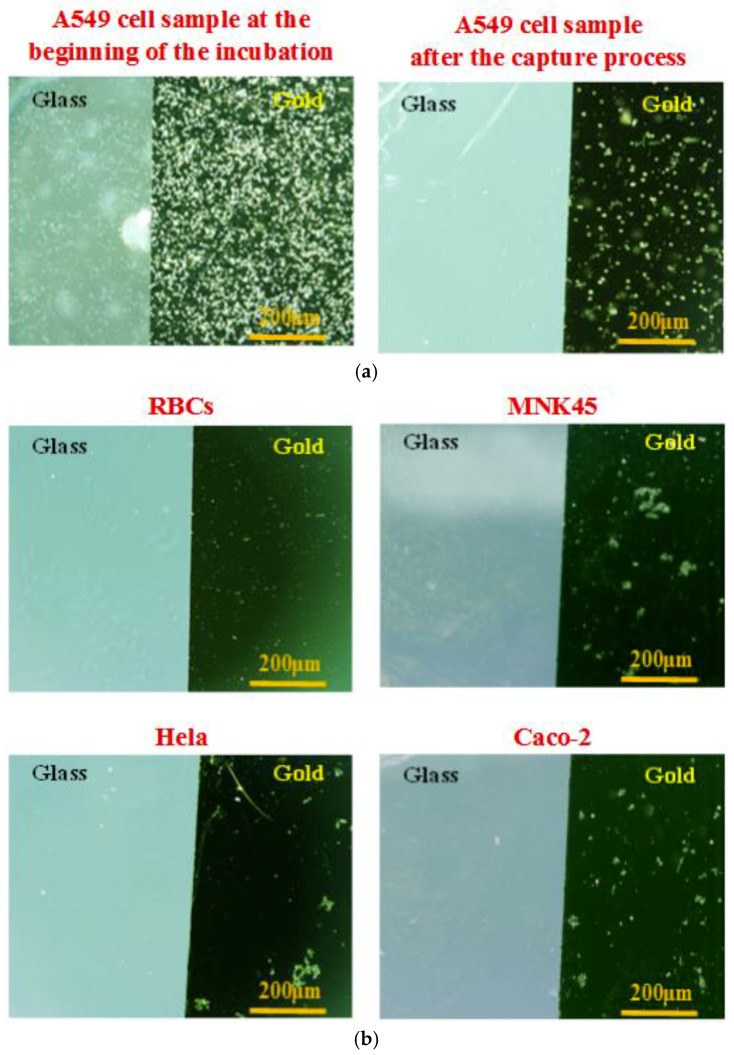
Microscopic images of the captured cells onto the aptamer-modified gold-glass substrates with different cell samples at the same cell concentration of 2 × 10^5^ cells/mL, and the same conditions of the capture process: (**a**) Target cell sample, human non-small cell lung cancer cells (A549 adenocarcinoma cells) at the beginning of the cell incubation and after the cell capture process. (**b**) Control cell samples at the end of the cell capture process consist of red blood cells (RBCs), human cervical cancer cells (Hela adenocarcinoma cells), human gastric cancer cells (MKN45 adenocarcinoma cells), and human colorectal cancer cells (Caco-2 adenocarcinoma cells).

Several investigations on the biochip were carried-out with various cell lines. In the current study, we compared the specificity among these human adenocarcinoma cells from various tissues including lung, cervix, stomach, and colon: A549 (human non-small cell lung cancer cells), Hela (human cervical cancer cells), MKN45 (human gastric cancer cells) and Caco-2 (human colorectal cancer cells). A549 cells were chosen as the target cells, whereas Hela cells, MKN45 cells, Caco-2 cells, and red blood cells (RBCs collected from volunteers) were the non-target cells used to evaluate the affinity and selectivity of the sensing probes. Each cell line sample was prepared at the same cell concentration of 2 × 10^5^ cells/mL. The cells captured onto the aptamer-modified gold electrodes surface were observed using the microscope. The pictures were taken at the beginning of the cell incubation and after the cell capture process. Microscopic images were obtained on the same objective scale, sensitivity, and exposure mode. The control parameters were selected by Olympus DP Controller image software. As seen in Figure 5, the device revealed a significantly high specificity toward A549 cells. A lot of A549 cells were captured stably, whereas nearly all the other cells were excluded by the aptamer-based gold surfaces. The findings indicated the significantly higher specificity of this aptamer-based capacitive sensing system for A549 isolation among these human adenocarcinoma cells.

These microscopic responses also demonstrated successful immobilization of the SAM layers onto the gold surface, and stable bonding of the aptamers to the SAM. In the practical surveys, various parameters that affect the biosensor response could be analyzed to establish the optimal conditions of the assay. In general, the response of the probes gradually enhanced with increasing the aptamer concentration. The results indicated that a stable state was reached at concentrations of aptamer of approximately 20 µM. The aptamer probes were normally operated in the media pH from 7.0 to 7.4 at room temperature. In addition, the response of the aptasensor gradually increased with increasing incubation time of cell samples, and reached stability at 1 min 30 s. However, a longer incubation time could lead to a higher number of cells being stuck onto the part of the glass surface. Thus, an incubation time of 90 s was chosen as the optimal incubation time of cell solution in subsequent experiments.

### 3.2. Impedance-Based Observations

**Figure 6:**
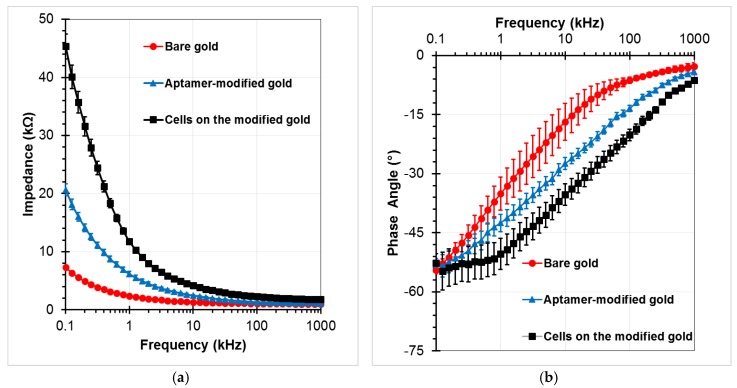
Impedance responses of the chip as a function of frequency for bare gold microelectrodes, aptamers-functionalized gold electrodes surface, and trapped A549 cells onto the modified electrodes with a voltage amplitude of 100 mV, and a frequency range from 100 Hz to 1 MHz: (**a**) Impedance magnitude, (**b**) Phase angle.

To confirm the SAMs generation procedure on the gold electrodes, the electrical impedance values between the sensing electrodes was measured in the PBS buffer medium at an amplitude of 100 mV, and the frequencies ranging from 0.1 kHz to 1 MHz. EIS responses were recorded at 40 points per decade. Fluid flow in the channel was stopped during impedance measurements. Three investigations were performed to compare the initial chip (bare gold), the chip after immobilization of aptamer (aptamer-modified gold), and the chip after incubation of target cells with the subsequent washing step (A549 cells on the aptamer-modified gold electrodes at the concentration of 2 × 10^5^ cells/mL). Figure 6 presents the measured impedance graphs in these three cases. Data were given in the form of amplitude Z (Figure 6a) and phase angle θ (Figure 6b). Each experimental data point represents the average value of at least three separate runs, and the error bar depicts the standard error of the mean. The impedance magnitude decreases as the frequency increases in the applied frequency range. The difference value at each frequency point increases up to tens of kilo Ohm between before and after standing of the aptamers onto the gold electrodes. The variations were observed more clearly at lower frequencies, especially after the cells were caught on the modified electrodes. The phase angle was close to −5° at the high frequency range of the impedance spectrum, whereas it approached −55° in the low frequency range. Therefore, the resistive element of impedance is dominant at the high frequency range, whereas the capacitance dominates the low frequency range where high magnitude changes can be observed. 

**Figure 7:**
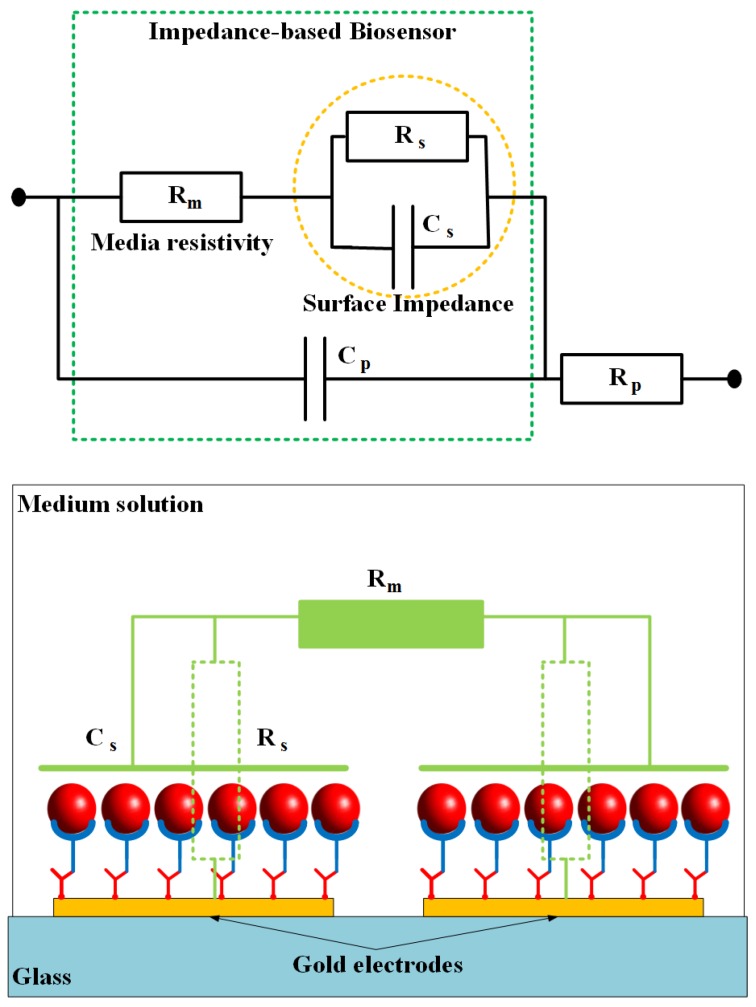
An electrical equivalent circuit model of biosensor based on impedance measurement method. The impedance is constructed of two main parts: surface impedance and resistance of salt media (PBS).

Figure 7 shows a simplified electrical equivalent circuit model of the impedance-based sensing platform where the microfluidic channel is fully filled by the buffer solution to create conductivity between the gold electrodes. The impedance of the biosensor consists of medium solution resistance *R_m_*, electrode-solution interface resistance *R_s_* and capacitance *C_s_*, and the parasitic capacitance *C_p_* and resistance *R_p_*. The parasitic resistance and capacitance are related to the connecting wires and the original electrode design. The other elements that depend on the conductivity and permittivity coefficients of the trapped cells, SAMs on the electrode surfaces, and the medium solution, are important parts of the sensor. By ignoring the parasitic impedance components, the total impedance *Z* of the sensor can be expressed by the following equation:

(1) $$Z\left(\omega \right)=R_{m}+\frac{R_{s}}{\omega^{2}R_{s}^{2}C_{s}^{2}+1}-\frac{\omega R_{s}^{2}C_{s}}{\omega^{2}R_{s}^{2}C_{s}^{2}+1}j$$

where, *ω* is the angle frequency, and *j* is the imaginary unit ($j^{2}=-1$). It can be seen that the impedance *Z* is approximate *R_m_* at high frequencies. Change in the total impedance mainly depends on the changes of the surface impedance at the low frequency range, in which the capacitance is the dominant component of the impedance. It is shown that the experimental data are in good agreement with the theoretical model. 

**Figure 8:**
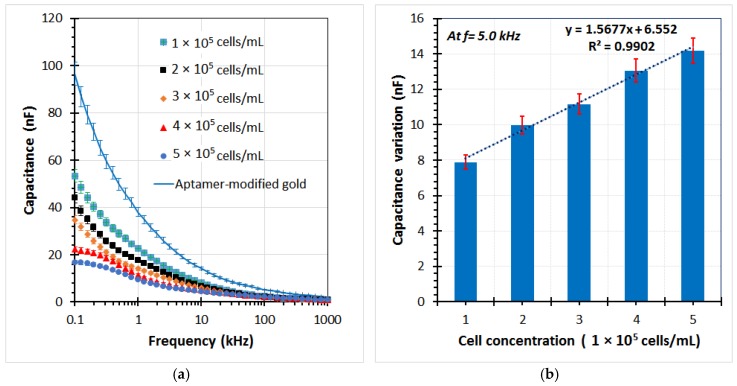
(**a**) Capacitance element of the impedance response for several A549 cell samples trapped on the aptamer-based sensing electrodes of the microchip by using the impedance analyzer at different cell concentrations, (**b**) The capacitance change of the chip with different cell concentrations respect to the aptamer-modified electrodes at frequency of 5 kHz.

Various A549 cell samples were used in the experiments to evaluate the performance of the sensor. The capacitance of the sensor was determined by the imaginary part of the impedance from the EIS data. Figure 8a shows the capacitance graphs at different cell concentrations ranging from 1 × 10^5^ to 5 × 10^5^ cells/mL. It can be seen that the capacitance magnitude decreased with an increase in cell concentration in the low frequency range. Subsequently, the capacitance change was calculated as the decrease of the capacitance value of each test sample in comparison with the chip without cells (the aptamer-modified gold electrodes). A highly linear relationship between the capacitance variation and the cell concentration was found at a reliable frequency of 5 kHz. The linear regression equation is expressed in Figure 8b, with the correlation coefficient (*R*^2^) up to over 99%. The limit of detection could be calculated from the formula 3σ/slope, where σ is the standard deviation; the slope is found from the linear response. Herein, a detection limit of the sensor was achieved at approximately 1.5 × 10^4^ cells/mL. In the current study, the main operating principles of the biochip using the aptamer-based assembly process on the gold electrodes for trapping target cells, and capacitance-based cell detection, have been expressed. The chip design, as well as the sensitivity of the sensor, can be further improved in subsequent works.

The experimental results revealed that the aptamers were successfully cultured on the gold substrate using the proposed functionalization method. Furthermore, EIS was proven to be a powerful and simple tool to demonstrate each step of modification of the electrode. The hand-held electrical measurement circuit board using cheap electronics components can be easily integrated with the sensor [37]. Thus, this method can be expressed more conveniently than other different approaches for the investigation of the immobilization of the aptamer. Other existing methods often require complex and expensive equipment, such as quartz crystal microbalance (QCM) [50], atomic force microscopy (AFM) [51], and surface plasmon resonance (SPR) measurements [52]. However, these methods are useful in early studies due to the potential to monitor cell–surface interactions, and affinity forces. In addition, in order to evaluate the storage stability of the sensing platform, aptamer-modified electrodes were stored in PBS buffer at 4 °C. After 15 days, EIS still maintained more than 90% of its initial signal response. The results indicated that the proposed sensor possesses an acceptable level of simplicity, rapidity, selectivity, and stability. In previous works, EGFR-bound A549 cells were captured by an electrode immobilized by anti-EGFR biomarker, and then the differential capacitance was read to detect their presence [49]. This study enables us to continuously develop a dielectrophoresis microfluidic enrichment chip combined with a highly sensitive impedance sensor for circulating tumor cell detection.

## 4. Conclusions

A simple and sensitive approach for detecting human lung carcinoma cells based on amine-terminated aptamer-modified gold microelectrodes was reported. An immobilization process onto the gold electrodes surface was proposed. The responses of the biosensor were examined by optical microscopic images and electrical impedance spectroscopy measurements. The equivalent circuit model for impedance-based detection was used to demonstrate the measured results. The sensor was confirmed to have high affinity against A549 cancerous cells as target cells compared with controls, i.e., RBCs, Hela cells, MKN45 cells, and Caco-2 cells. The detection sensitivity of the sensor for A549 cells was evaluated through the measurement of capacitance variation. A higher detection efficiency of the sensor was observed at a frequency of 5 kHz. A linear relationship was found between the capacitance variation and cell concentration in the range from 1 × 10^5^ to 5 × 10^5^ cells/mL, with the correlation coefficient up to 99%. Although the detection capacity of the current sensor was still limited, the biochip exhibited many attractive features, namely, simplicity, rapidity, low-cost, biocompatible, selectivity, and sensitivity toward the diagnosis of lung cancerous cells. The electrode design, as well as the impact parameters of the proposed method, should be continuously optimized for cancerous cell quantification. Meanwhile, the electronic measurement circuit module for the sensor can be further developed.
